# AMRx Hub: a curated web platform for discovery of antimicrobial resistance resources

**DOI:** 10.1128/mra.00997-26

**Published:** 2026-10-26

**Authors:** Elijah Kolawole Oladipo, Akinloluwa Isaac Adeolu-Shittu, Adeniyi Daniel Fatuyi-Adene, Joy Olamiposi Emmanuel

**Affiliations:** 1 Division of Vaccine and Pharmacotherapies Design and Development, Helix Biogen Institute 674962, Ogbomoso, Oyo State, Nigeria; 2 Division of Genome and Molecular Sciences, Helix Biogen Institute 674962, Ogbomoso, Oyo State, Nigeria; 3 Division of Diagnostics Assay Development and Medical Artificial Intelligence, Helix Biogen Institute 674962, Ogbomoso, Oyo State, Nigeria; 4 Department of Microbiology, Laboratory of Molecular Biology, Immunology and Bioinformatics, Adeleke University 385809, Ede, Osun State, Nigeria; 5 Department of Chemical Engineering, University of Birmingham 1724, Birmingham, United Kingdom; 6 Department of Biological Sciences, Redeemer's University, Ede, Osun State, Nigeria; 7 Department of Microbiology, Federal University of Technology, Akure, Ondo State, Nigeria; 8 Department of Microbiology, Kwara State University 269039, Ilorin, Kwara State, Nigeria; University of Michigan, Ann Arbor, Michigan, USA

**Keywords:** AMR, repository, One Health, web interface

## Abstract

AMRx Hub is an open-access web platform developed to address the fragmentation of antimicrobial resistance (AMR) resources by aggregating AMR-related resources into a centralized searchable repository, supporting researchers, educators, and policymakers through a One Health framework.

## ANNOUNCEMENT

Antimicrobial resistance (AMR) remains one of the most pressing global public health challenges, threatening the effectiveness of antimicrobial therapies and increasing morbidity, mortality, and healthcare costs worldwide (1). Although numerous AMR-related databases, bioinformatics tools, surveillance systems, educational resources, and policy documents exist, these resources are frequently dispersed across governmental agencies, research institutions, international organisations, and independent platforms, making discovery and utilisation sometimes challenging for end users (2, 3).

**Fig. 1:**
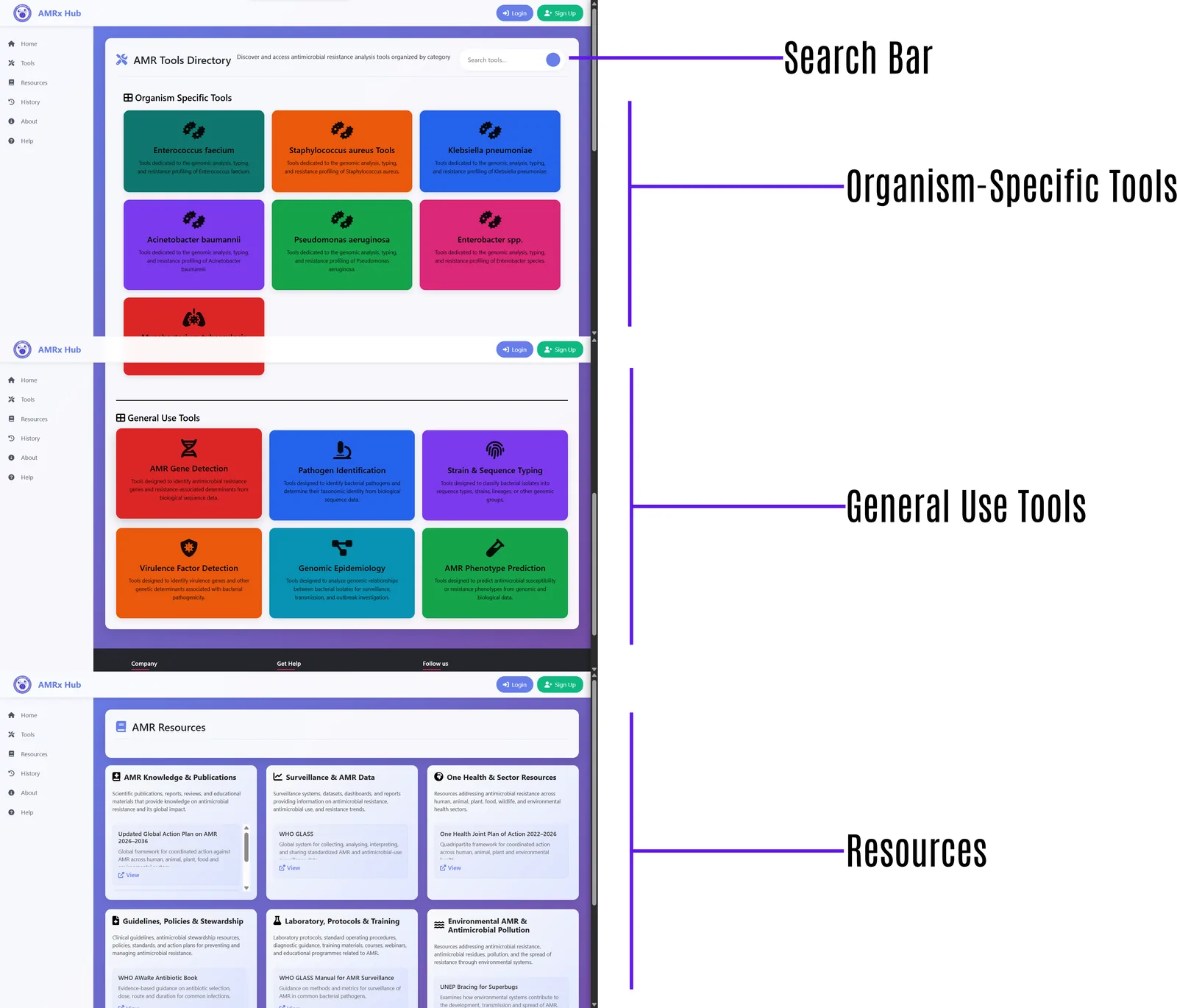
AMRx Hub web interface showing a search bar and the organization of antimicrobial resistance resources into organism-specific tools, general-use tools, and resources.

AMRx Hub is designed around the One Health framework, connecting users with computational tools, databases, resources, and information systems for AMR research across human, animal, and environmental health (4, 5). It provides a centralized, curated, and searchable repository that improves resource accessibility and reduces fragmentation (Fig. 1). Organism-specific tools currently cover all six ESKAPE pathogens (*Enterococcus faecium*, *Staphylococcus aureus*, *Klebsiella pneumoniae*, *Acinetobacter baumannii*, *Pseudomonas aeruginosa*, and *Enterobacter* spp.) and *Mycobacterium tuberculosis*, alongside general-use tools grouped by analytical function. The platform is freely accessible without login or registration, allowing users to explore AMR resources openly (6). Registered users can save resources for future access and view a history of the tools they have accessed.

AMRx Hub uses structured metadata to index resources across multiple categories, enabling efficient search and discovery. Records include names, categories, institutions, authors, versions, licenses, descriptions, features, requirements, logos, and screenshots, with direct links to external resources. These metadata can be accessed by clicking the information icon at the top right corner of the tool description box. Table 1 shows examples of tools linked through AMRx Hub (6).

**TABLE 1:** Examples of tools linked through AMRx Hub

Tool	Category	Description	References
PubMLST *Staphylococcus aureus*	*Staphylococcus aureus*	The definitive primary repository and web-based typing platform for the classic seven-gene *Staphylococcus aureus* MLST scheme.	(7)
*Pseudomonas* Genome Database	*Pseudomonas aeruginosa*	A comprehensive web database providing curated genomic data, comparative analysis tools, and annotations for *Pseudomonas* species.	(8)
Resistance Gene Identifier (RGI)	AMR Gene Detection	Web-based implementation of Resistance Gene Identifier (RGI) for identifying antimicrobial resistance determinants from nucleotide or protein sequences using the curated CARD database.	(9)
EnteroBase	Genomic Epidemiology	Web-based platform for bacterial genomic epidemiology, genome assembly, sequence typing, hierarchical clustering, and epidemiological analysis.	(10)

Future tools and resources will be considered for inclusion according to the AMRx Hub taxonomy system, a standardized framework that defines the criteria, metadata requirements, and classification rules used to evaluate and organize resources within the Hub. The taxonomy is intended to promote consistency in resource selection and categorization. Currently, designated AMRx Hub administrators and staff are responsible for reviewing resources and have final authority to edit, approve, and add them to the platform. Resource governance and approval authority will remain subject to the AMRx Hub’s defined administrative framework.

AMRx Hub is an operating system independent and accessible through modern web browsers without local installation. Documentation and user support materials are continuously updated to facilitate adoption. Future priorities include automated link validation, enhanced search, community resource submissions, metadata standardization, expanded AMR coverage, AI-assisted curation, and integration with additional repositories. The development team commits to maintaining the platform, source code, and documentation for at least 5 years following publication.

## Data Availability

AMRx Hub version 0.9.5 is publicly accessible at https://amrxhub.helixbiogeninstitute.org/. Source code is available at https://github.com/ender-ma/AMRxHub. The DOI of the project can be accessed here: https://doi.org/10.5281/zenodo.22180074. The repository includes installation instructions, documentation, and licensing information under the Apache-2.0 license, an open-source license. Significant platform functionality is accessible without user registration. Available documentation can be accessed here: https://amrxhub.helixbiogeninstitute.org/documentation/ and tutorial materials are here: https://amrxhub.helixbiogeninstitute.org/help/. These materials will be updated regularly.

## EDITOR'S NOTE

**Ed Note**: This article was handled by Pat Schloss, who acted as a guest editor in consultation with the editor in chief, Irene Newton. Dr. Schloss's role as the guest editor of this article is outside of his duties as the chair of the Journals Committee.
